# Supplementary material for: EMS-Induced Mutagenesis of Clostridium carboxidivorans for Increased Atmospheric CO2 Reduction Efficiency and Solvent Production
Source: Microorganisms. 2020 Aug 14;8(8):1239. doi: 10.3390/microorganisms8081239 (PMC7464951; doi:10.3390/microorganisms8081239)
Supplement: Supplementary file 1 [file microorganisms-08-01239-s001.pdf]

EMS-induced mutagenesis of *Clostridium carboxidivorans* for increased atmospheric CO_2 reduction efficiency and solvent formation

Naoufal Lakhssassi[1,2], Azam Baharlouei[1], Jonas Meksem[3], Scott D. Hamilton-Brehm[4], David A. Lightfoot[2], Khalid Meksem[2*], and Yanna Liang[1,5*]

[1]Department of Civil and Environmental Engineering, 1230 Lincoln Drive, Southern Illinois University Carbondale, Carbondale, IL 62901, USA
[2]Department of Plant, Soil, and Agricultural Systems, Southern Illinois University, Carbondale, IL 62901, USA.
[3]Duke University, Durham, NC 27708, USA.
[4]Department of Microbiology, Southern Illinois University, Carbondale, IL 62901, USA.
[5]Department of Environmental and Sustainable Engineering, 1400 Washington Ave, State University of New York at Albany, Albany, NY 12222, USA
*Corresponding authors, E-mail address: yliang3@albany.edu (for HPLC Phenotyping) and meksem@siu.edu (for EMS mutagenesis and mutant development).

Supplemental information

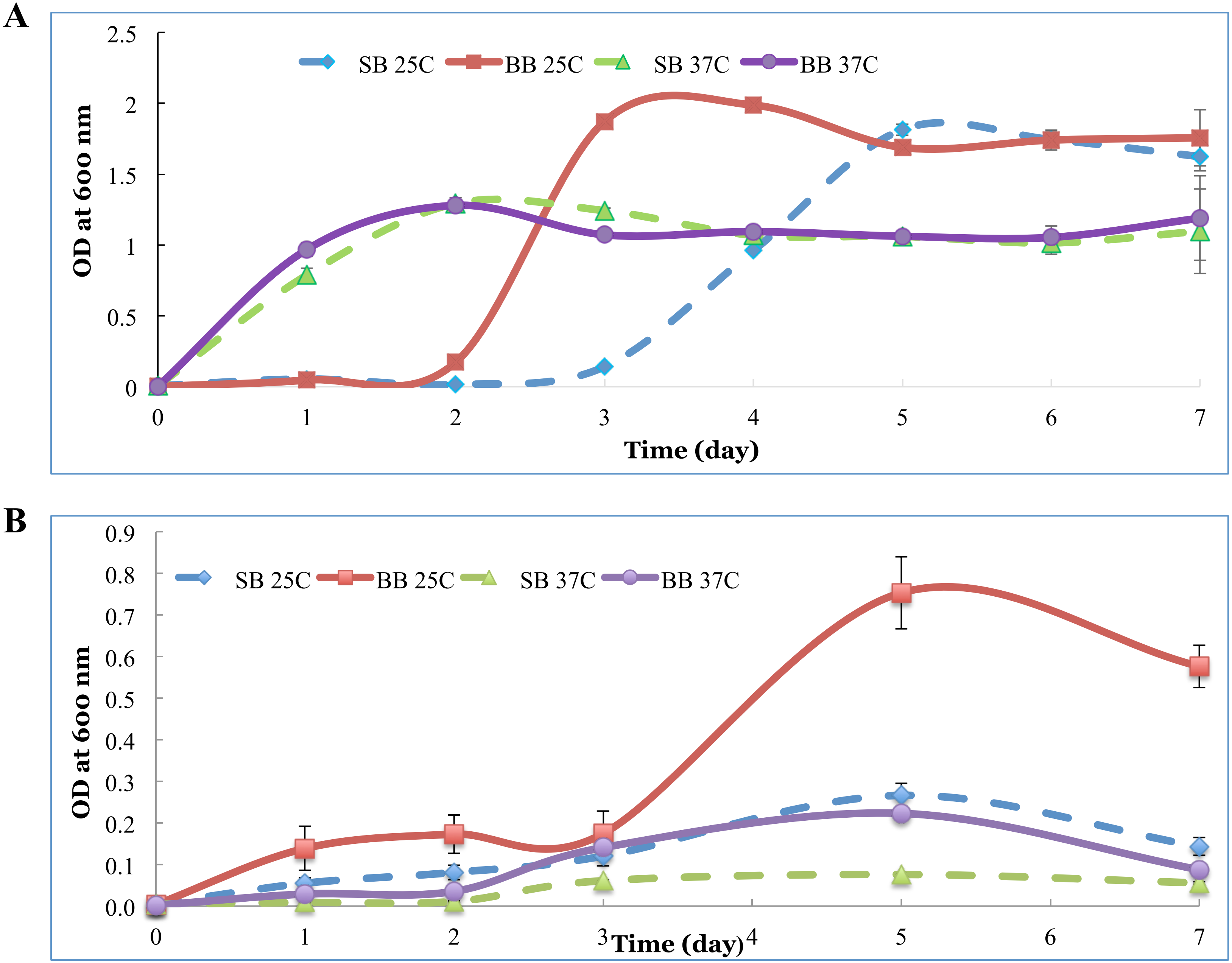

Materials: Supplemental Figure S1. Comparison of growth of the wild type P7 in the WC medium **(A)** and the 1754-B medium **(B)**. The P7 performed better at 25 °C than at 37 °C in both WC and 1754-B medium.

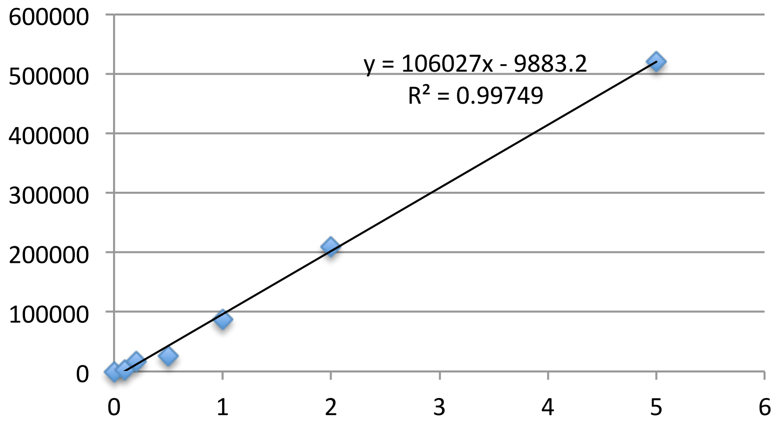

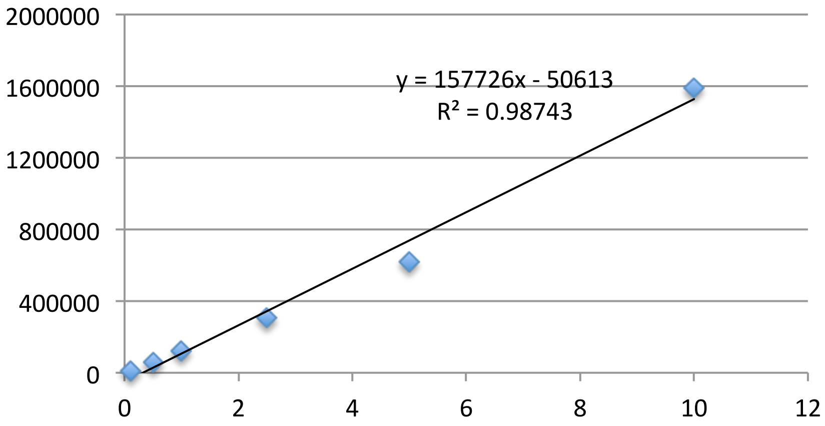

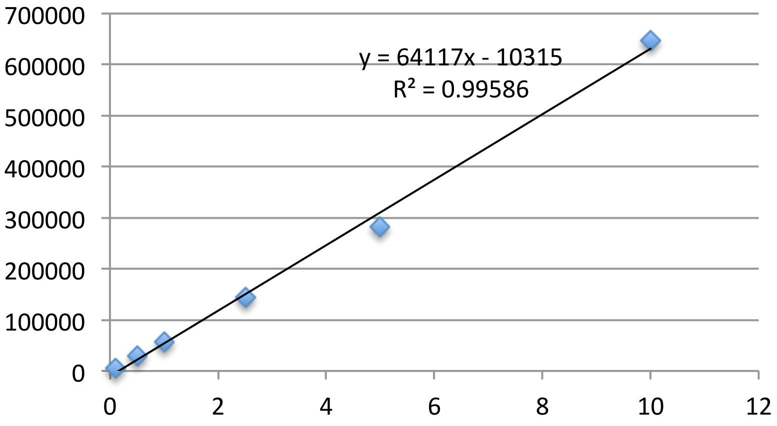

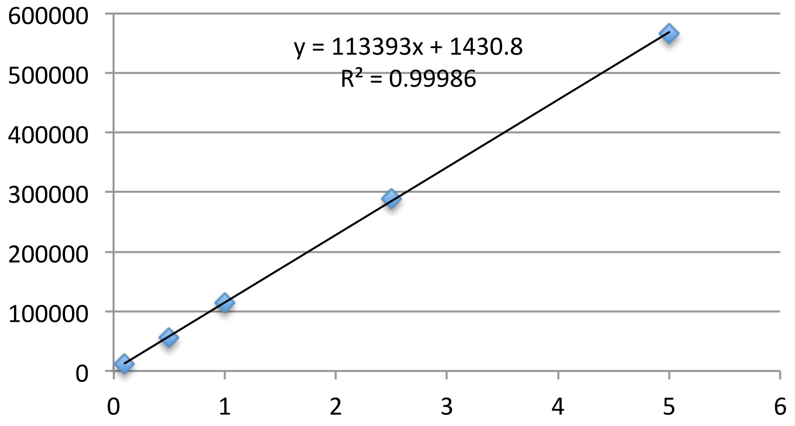

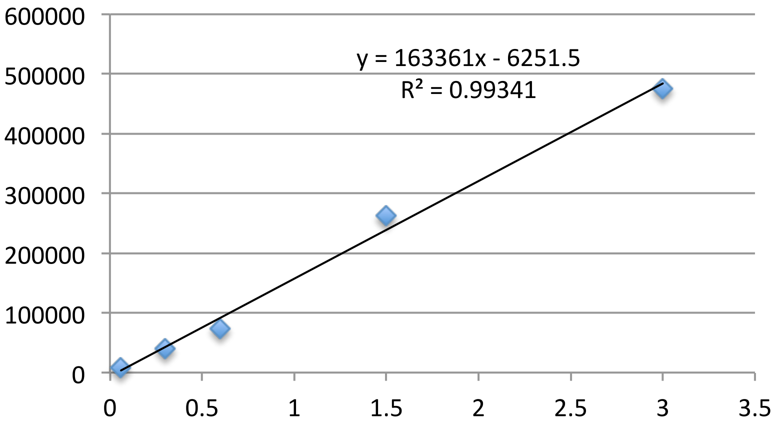

Supplemental Figure S2. Calibration curves of (A) Formic acid, (B) Acetic acid, (C) Ethanol, (D) Butyrate acid, and (E) Butanol. Equation and R2 are shown, (*R>0.99*). Equations were used to calculate the P7-WT and P7-EMS mutant end products corresponding to each retention time detected by HPLC. The X-axis corresponds to the different concentrations used, the Y-axis corresponds to the peak area detected by HPLC.

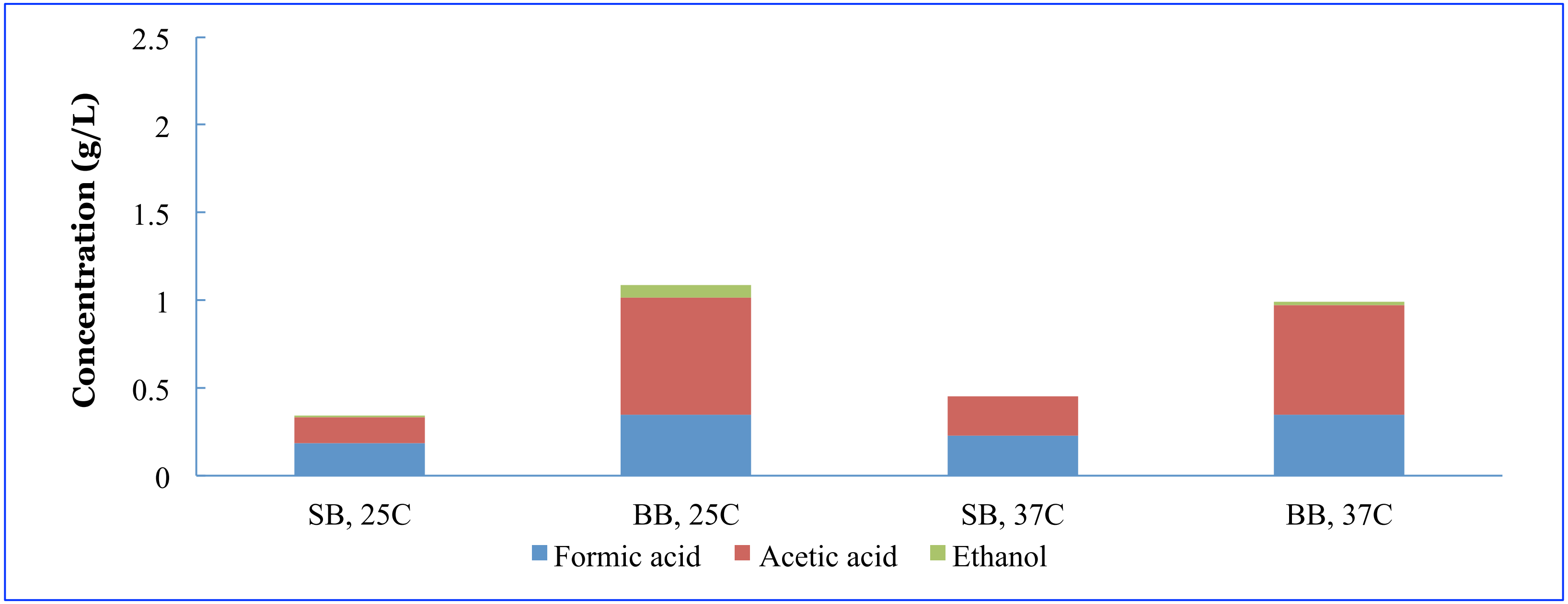

Supplemental Figure S3. Comparison between the end products identified for the wild type P7 when grown in the 1754-B defined medium on day 7.
